# Magnitude and breadth of antibody cross-reactivity induced by recombinant influenza hemagglutinin trimer vaccine is enhanced by combination adjuvants

**DOI:** 10.1038/s41598-022-12727-y

**Published:** 2022-06-02

**Authors:** Jenny E. Hernandez-Davies, Emmanuel P. Dollinger, Egest J. Pone, Jiin Felgner, Li Liang, Shirin Strohmeier, Sharon Jan, Tyler J. Albin, Aarti Jain, Rie Nakajima, Algimantas Jasinskas, Florian Krammer, Aaron Esser-Kahn, Philip L. Felgner, Qing Nie, D. Huw Davies

**Affiliations:** 1 Vaccine Research and Development Center, Department of Physiology and Biophysics, School of Medicine, University of California, Irvine, CA 92697 USA; 2 Department of Mathematics, University of California, Irvine, CA 92697 USA; 3 Department of Microbiology, Icahn School of Medicine at Mount Sinai, New York, NY 10029 USA; 4 Department of Chemistry, University of California, Irvine, CA 92697 USA; 5 Department of Pathology, Molecular and Cell-Based Medicine, Icahn School of Medicine at Mount Sinai, New York, NY 10029 USA; 6 Pritzker School of Molecular Engineering, University of Chicago, Chicago, IL 60637 USA; 7 Present Address: Avidity Biosciences, San Diego, CA 92121 USA

**Keywords:** Immunology, Vaccines, Adjuvants

## Abstract

The effects of adjuvants for increasing the immunogenicity of influenza vaccines are well known. However, the effect of adjuvants on increasing the breadth of cross-reactivity is less well understood. In this study we have performed a systematic screen of different toll-like receptor (TLR) agonists, with and without a squalene-in-water emulsion on the immunogenicity of a recombinant trimerized hemagglutinin (HA) vaccine in mice after single-dose administration. Antibody (Ab) cross-reactivity for other variants within and outside the immunizing subtype (homosubtypic and heterosubtypic cross-reactivity, respectively) was assessed using a protein microarray approach. Most adjuvants induced broad IgG profiles, although the response to a combination of CpG, MPLA and AddaVax (termed ‘IVAX-1’) appeared more quickly and reached a greater magnitude than the other formulations tested. Antigen-specific plasma cell labeling experiments show the components of IVAX-1 are synergistic. This adjuvant preferentially stimulates CD4 T cells to produce Th1>Th2 type (IgG2c>IgG1) antibodies and cytokine responses. Moreover, IVAX-1 induces identical homo- and heterosubtypic IgG and IgA cross-reactivity profiles when administered intranasally. Consistent with these observations, a single-cell transcriptomics analysis demonstrated significant increases in expression of IgG1, IgG2b and IgG2c genes of B cells in H5/IVAX-1 immunized mice relative to naïve mice, as well as significant increases in expression of the IFNγ gene of both CD4 and CD8 T cells. These data support the use of adjuvants for enhancing the breath and durability of antibody responses of influenza virus vaccines.

## Introduction

Despite seasonal vaccination, influenza virus remains a major source of annual morbidity and mortality, and a constant pandemic threat. Influenza A virus (IAV) evolves as it replicates in human and animal hosts owing to an error-prone RNA polymerase that lacks proof-reading function, randomly generating amino acid substitutions in viral proteins. Under selection by the immune response, mutants emerge that escape existing immunity^1–4^. The major target of neutralizing antibodies (nAbs) elicited by infection or vaccination is hemagglutinin (HA) on the virion surface, which functions on the virion as a receptor for cell-surface sialic acid to mediate viral entry into host cells^5,6^. Thus far, 16 distinct HA subtypes have been found in IAV isolated from mammalian and avian hosts, and two more from bats^7,8^. A secondary target of nAbs is neuraminidase (NA), a viral-surface enzyme that facilitates egress from infected cells. There are 11 known NA subtypes. Immune escape is caused by the accumulation of amino acid substitutions in epitopes recognized by nAbs, a process known as antigenic drift^3,9,10^. This process has led to the emergence of many more drifted variants of HA than of NA.

The segmented RNA genome of influenza virus can also lead to the wholesale exchange of different HA subtypes in cells co-infected with two or more different subtypes of influenza virus^11^, a process known as antigenic shift. Currently only IAVs expressing H1 and H3 subtypes are circulating in humans. However, exchange with a novel HA subtype to which humans have not been previously exposed has the potential to cause pandemics. For example, the highly pathogenic avian influenza virus H5N1 A/goose/Guangdong/1996 lineage is endemic in poultry in several countries and has caused sporadic lethal infections in humans^12^. The antigenic novelty of these viruses pose a continued risk for pandemic potential in naïve human populations^13,14^. Pre-pandemic preparedness involves stockpiling vaccines against potential pandemic IAVs. Ultimately, however, it is unknown which subtype will lead to the next pandemic. Thus, the induction of broad protection is as important for pandemic vaccines as it is for seasonal vaccines.

Protection afforded by seasonal vaccination is short-lived, necessitating revision of the vaccine strains on an annual basis. Effectiveness against circulating strains varies year-to-year, anywhere from 0 to 60%, depending on the accuracy with which the vaccine strains predicted correspond to the strains that are eventually seen in circulation^15^, and the overall immune competence of the vaccinee^16,17^. Both of these limitations conspire to reduce vaccine efficacy and durability, which ultimately leads to varying levels of morbidity and mortality in susceptible populations. It is likely effectiveness could be improved by a broadly-protective seasonal vaccine which targets conserved epitopes shared by variants^18–21^.

In this study, we administered mice a recombinant trimerized HA (either H5 from A/Vietnam/1203/04 or H7 from A/Anhui/1/2013) with 8 different toll-like receptor (TLR) agonists, with or without an oil-in water emulsion, to assess immunogenicity and breadth of the response. All of the adjuvants tested enhanced these responses over antigen alone to varying levels and with different Th1/Th2 ratios. A combination adjuvant we refer to as “IVAX-1”, comprising synthetic oligodeoxynucleotides containing unmethylated cytosine-guanine motifs (CpG ODNs) and monophosphoryl lipid A (MPLA) (TLR9 and 4 agonists, respectively) with emulsion induced broad Ab responses by microarray that developed quickly and reached greatest magnitude. The response was skewed towards Th1 (IgG2c>IgG1) and included nAbs after boosting. A single-cell transcriptomic analysis of whole spleens from mice receiving HA/IVAX-1 confirmed the polarization seen by immunological methods, with additional insights into the spectrum of effects of IVAX-1 during a virus neutralizing response.

## Materials and methods

### Antigens and viruses

Trimerized HA from A/Vietnam/1203/04 (H5N1) or A/Anhui/1/2013 (H7N9) were expressed in insect cells and purified on nickel chelate columns as described^22^. Full-length HA0 and HA1 regions for protein microarray printing, B cell labeling and T cell recall assays, were purchased from Sino Biological Inc. (Beijing, China). Reassortant influenza virus A/Vietnam/1194/2004 (H5N1) × A/Puerto Rico/8/1934 (NIBRG-14 from National Institute for Biological Standards & Controls, South Mimms, UK) was propagated in fertilized hen eggs (Charles River) and titers determined by hemagglutination^23^ and TCID_50_ assay, where TCID_50_ is the tissue-culture infective dose, i.e., dilution of virus that caused a cytopathic effect in 50% of wells containing Madin-Darby canine kidney (MDCK) cells^24^. All virus handling was performed in USDA inspected and approved BSL2+/ABSL2+ facilities.

### Adjuvants

Tri-agonist TLR2/6_4_7 was synthesized by conjugation reactions (amide bond formation, maleimide-thiol Michael addition and azide-alkyne click chemistry) of individual TLR agonists to a central triazine as described previously^25^. The individual agonists comprised Pam_2_CSK_4_, a TLR2 and TLR6 agonist (TLR2/6 a), pyrimido-indole (TLR4 a), and 2Bxy, an imidazoquinoline derivative (TLR7 a). TLR tri-agonist and individual TLR agonists were purified by either high performance liquid chromatography or gel extraction, and their masses were confirmed by matrix-assisted laser desorption/ionization-time of flight (MALDI-TOF) or electrospray ionization-mass spectrometry. The CpG-1018 (TLR9 a) was purchased from Integrated DNA Technologies (Coralville, Iowa) and dissolved in sterile water at 1 mM as stock. Monophosphoryl lipid A (MPLA, TLR4 a) was purchased from Avanti Polar Lipids Inc., (Alabaster, AL; purity > 99%). Since MPLA has limited solubility in aqueous solution, this agonist was integrated into liposomes of the inert colipid 1,2-dioleoyl-sn-glycero-3-phospho-(1′-rac-glycerol) (DOPG; Avanti Polar Lipids Inc., Alabaster, AL) an at 1:5 molar ratio. A squalene oil-in-water emulsion (AddaVax) was purchased from Invivogen Inc. (San Diego, CA). Absence of endotoxin (endotoxin activity of < 1 EU/mL) in TLR a samples, with the exception of MPLA, was confirmed with LAL Endotoxin Assay Kits (Genscript, Piscataway, NJ) or by HEK TLR4 reporter cell assay (Invivogen, San Diego, CA). MPLA is manufactured in organic solvents and has minimal endotoxin activity.

### Mouse immunizations

All animal work was approved by the UCI Institutional Animal Care and Use Committee (IACUC protocol No. AUP-18-096) and by the Animal Care and Use Review Office (ACURO) of the U.S. Army Medical Research and Materiel Command (USAMRMC). The laboratory animal resources at UCI are Internationally accredited by the Association for Assessment and Accreditation of Laboratory Animal Care (AAALAC No. 000238). All experiments were performed in accordance with the animal use protocol approved by IACUC and ACURO. This article also follows recommendations in the ARRIVE guidelines for reporting of animal research^26^. Female C57Bl/6 mice (7–10 weeks of age) were purchased from Charles River Inc., and housed in standard cages with enrichment. Each vaccine dose comprised 2.5 μg trimerized HA antigen with different TLR agonists (Table 1). Formulations were used without or with an equal volume of AddaVax and administered via the sub-cutaneous (s.c.) route (50 μL, base of tail) under brief anesthesia with inhaled isofluorane/O_2_ mixture. Mice were weighed and monitored daily for the first two weeks for any changes in behavior or appearance and periodically thereafter. Blood was collected into heparinized microcapillary tubes at regular time points (d0, d10, d28, d43) by facial vein bleed under anesthesia with inhaled isofluorane/O_2_ and at the experimental endpoint (d103) by cardiac puncture under terminal anesthesia. Tissues (spleens, lymph nodes) were harvested post-mortem for T cell recall assays. For intranasal immunizations (i.n.), vaccine formulations containing 2.5 μg trimerized H5 were administered in a total volume of 20 μL using a micropipette pipette to the right-side nares of mice transiently anesthetized in an isofluorane/O_2_ mixture. These mice were boosted via the same route and dose on d56 and lung and nasal washes collected 13 days later.

**Table 1:** Formulations used for adjuvant screen.

Group	Antigen	TLR agonist						Emulsion
	H5 trimer (μg)	TLR2/6_4_7 (nmole)	TLR2/6 (nmole)	TLR4 (PID) (nmole)	TLR7 (nmole)	TLR9 (CpG) (nmole)	TLR4 (MPLA) (nmole)	AddaVax (μL)
1	2.5	1						
2	2.5		1	1	1			
3	2.5		1					
4	2.5			1				
5	2.5				1			
6	2.5					1		
7	2.5						3	
8	2.5					1	3	
9	2.5							
10	2.5	1						25
11	2.5		1	1	1			25
12	2.5		1					25
13	2.5			1				25
14	2.5				1			25
15	2.5					1		25
16	2.5						3	25
17	2.5					1	3	25
18	2.5							25

Groups of female C57Bl/6 mice (N = 4 per group) were administered antigen and TLR agonist(s) to a final volume of 50 μL in PBS per mouse (groups 1–9) or 25 μL in PBS and an equal volume of AddaVax emulsion added (groups 10–18), and administered via the s.c. route (base of tail).

### Protein microarrays

HA protein microarrays were fabricated as previously described^27^. Briefly, purified influenza HAs spanning 18 influenza A virus subtypes expressed in human or insect cells as either HA0 or HA1 molecules, were purchased from Sino Biological Inc. (Beijing, China). Each lyophilized antigen was reconstituted to a concentration of 0.1 mg/mL in phosphate-buffered saline (PBS) with 0.001% Tween 20 (T-PBS) and printed onto nitrocellulose-coated glass AVID slides (Grace Bio-Labs, Inc., Bend, OR, USA) using an Omni Grid 100 microarray printer (Genomic Solutions). For probing, plasma samples were diluted 1:100 in protein array blocking buffer (GVS, Sanford, ME, USA) supplemented with a poly-histidine peptide (HHHHHHHHHHGGGG) (Biomatik USA, Delaware, USA) to final concentration 0.10 μg/mL, and pre-incubated at room temperature (RT) for 30 min. Concurrently, arrays were rehydrated in blocking buffer for 30 min prior to replacement of the blocking buffer with pre-incubated plasma. Arrays were incubated overnight at 4 °C with gentle agitation. After washing 6 times with Tris-buffered saline (TBS) containing 0.05% Tween 20 (T-TBS) at RT, arrays were incubated for 2 h at RT with biotinylated anti-mouse IgG (Jackson ImmunoResearch; Cat No. 115-068-071) or anti-mouse IgA (Kirkegaard & Perry Laboratories [KPL]; Cat. No. 16-18-01). Arrays were then washed 6 times with T-TBS, followed by incubation with streptavidin-conjugated Qdot-800 (Life technologies; Cat. No. Q10173MP) diluted 1:250 in blocking buffer for 1 h at RT. For IgG subtyping, anti-mouse IgG1-Alexa Fluor647 or IgG2c-Alexa Fluor555 (Southern Biotech; Cat. Nos. 1073-31 and 1077-32) were used. Arrays were washed three times each with T-TBS followed by TBS, dipped in distilled water, and air-dried by centrifugation at 500 × *g* for 10 min. Images were acquired using the ArrayCAM imaging system (Grace Bio-Labs Inc., Bend, OR) with gain and exposure times of 50 and 400–1000 ms, respectively. Spot and background intensities were measured using an annotated grid (.gal) file. Signal intensities (SI) for each antigen on the array were first background corrected by subtracting sample-specific T-PBS buffer signals from purified protein spot signals.

### Hemagglutination inhibition (HI) assays

HI assays were performed essentially as described^23^. Mouse plasma samples were tested in duplicate by first treating 10 μL plasma with 30 μL receptor destroying enzyme (RDE; Denka Seiken, Inc.) for 18 h at 37 °C, after which 30 μL of 2.5% sodium citrate was added and heated at 56 °C for 30 min^24^. The volume was brought up to 100 μL with PBS, pH7.2 to give a starting dilution of 1/10. Duplicate serial dilutions of 25 μL plasma/well were performed across a V-bottomed microtiter plate (Thermo Fisher) in HI assay buffer (FTA Hemagglutination Buffer, Fisher Scientific) and an equal volume of virus (diluted to 4 hemagglutination units [HAU]/25 μL HI assay buffer) added for 30 min at RT to allow neuralization to occur. One row without plasma or virus (HI assay buffer only) and one row of without plasma (virus only) were also included as controls. To each well was added 50 μL 0.5% freshly-washed turkey red blood cell suspension (Rockland Immunochemicals, Inc., Limerick, PA) in HA buffer and left for 45 min at 4 °C to allow red cell pellets to form. The neutralization titer was defined as the reciprocal of the last dilution in which a clear pellet was seen, and multiplied by 10 to correct for the initial dilution from RDE treatment. An HI titer of 1/40 is considered the cut-off for protection in humans^28^. Positive control plasma for neutralization assays were produced against reassortant H5N1 virus (obtained from NIBSC, UK, Cat. No. NIBRG-14) in C57Bl/6 mice by administering 5 × 10^4^ TCID_50_ in 10 μL via i.n. route followed 2 weeks later by the same dose administered in 100 μL via intraperitoneal (i.p.) route.

### Microneutralization (MN) assays

MN assays were performed as described^24^ with modifications. Briefly, MDCK cells were obtained from ATCC (Manassas, VA; Cat. No. CCL-34) and maintained in Eagle’s minimum essential medium (EMEM; ATCC Cat. No. 30-2003) containing penicillin/streptomycin (Thermofisher) and 10% heat inactivated fetal calf serum (ATCC-Cat. No. 30-2020), and cultured at 37 °C/5% CO_2_ in a humid environment. Cells were sub-cultured when 80–85% confluency and only early passage cells were used for MN assays. One day prior to assay, MDCK cells were sub-cultured into flat-bottomed 96 well plates at 2 × 10^4^ cells/well in 100µL. Serum samples were treated overnight with 3 volumes of RDE for 18 h at 37 °C, and then inactivated at 56 °C for 30 min. Plasma were then diluted 1:10 in virus growth media, comprising serum-free EMEM containing 0.6% BSA and 1 µg/mL N-p-Tosyl-l-phenylalanine chloromethyl ketone (TPCK)-treated trypsin (Worthington Biochemical), and two-fold serially diluted in virus growth medium in a separate 96-well plate. Reassortant H5N1 virus (NIBSC; Cat. No. NIBRG-14) was diluted to 100 TCID_50_ per 50 µL in virus growth media, and 50 µL of virus added to wells containing the serially diluted plasma, and incubated for 1 h at 37 °C/5% CO_2_. Control wells contained virus only and growth media only. After 1 h incubation, media from cell monolayers was replaced with the serum-virus mixtures and plates incubated for 1 h. Serum-virus mixtures were then replaced with 200 µL of virus growth media + 2% fetal bovine serum, and plates incubated for 48 h at 37 °C. Cells were then fixed in 4% paraformaldehyde (Fisher Scientific; Cat. No. AAJ19943K2) in PBS for 30 min, washed in PBS and permeabilized in 0.1% PBS/Triton X-100 at RT for 15 min. Cells were then washed and blocked in a blocking buffer of 3% bovine serum albumen (BSA; Sigma-Aldrich, Cat. No. A9085) in PBS for 1 h at RT. Influenza virus nucleoprotein (NP) was then detected using anti-NP mAbs (Millipore Cat Nos. MAB 8257 and MAB 8258) combined at 1/1000 dilution each in blocking buffer, followed by horse radish peroxidase (HRP)-conjugated anti-mouse IgG (KPL, Cat. No. 074-1802) diluted to 1:3000 in blocking buffer. Plates were developed in 3,3′,5,5′-tetramethylbenzidine (TMB) peroxidase substrate (SureBlue; SeraCare KPL Cat. No. 5120-0075) and reactions stopped using 0.18 M H_2_SO_4_. Assays were quantified in an enzyme-linked immunosorbent assay (ELISA) plate reader (Emax Plus, Molecular Devices LLC, San Jose, CA) at 450 nm using SoftMax Pro 7.1 software.

### T cell recall assays

Mice that had been immunized with trimerized H5 VN04 for serological studies were re-used for T cell recall assays at the serological endpoint. Prior to the assay, mice were boosted via the i.p. route with 100 μL the same vaccine formulation used to prime, and splenocytes were harvested 10 days later. Recall assays were performed using ELISpot format essentially as described previously^29^. Antigens used for recall were HAs expressed in HEK293 cells (Sino Biological: H1 Cal09, Cat. No. 11055-V08H; H5 VN04 Cat. No. 11062-V08H2; and H7 AH13 Cat. No. 40103-V08H) as well as Ebola virus glycoprotein expressed in insect cells (IBT BioServices Cat. No. 0501-015). Assays were performed in T cell medium (TCM) comprising Iscove’s Modified Dulbecco’s Medium (IMDM), containing 5 × 10^-5^ M β-mercaptoethanol, 100 IU/mL penicillin, 100 μg/mL streptomycin, and 10% fetal calf serum as described^29^. After 18 h of incubation, the assay supernatants were collected for multiplex cytokine screening using the LEGENDplex kit (BioLegend Inc., San Diego, CA) according to the manufacturer’s instructions, before the ELISpot was processed. T cell recall assays were also performed using virus-infected antigen-presenting cells (v-APCs) as a source of recall antigen for restimulating CD8 T cells. For this, erythrocyte-depleted splenocytes were washed 3× in PBS, then incubated with H5N1/PR8 reassortant influenza virus to a final concentration of 1 HAU in serum-free infection medium (Eagles Minimal Essential Medium, 0.6% BSA, 1 μg/mL EDCP-treated trypsin + antibiotics) at 37 °C/5%CO_2_ with gentle agitation every 10 min. After 1 h incubation, v-APCs were washed 1× in PBS then resuspended at 10^7^ cells/ml in TCM (as described above) containing 0.2% FCS. The v-APCs were used neat (10^7^ cells/ml) or diluted to 10^6^, 10^5^ and 0 cells/mL in a suspension of 10^7^ cells/mL uninfected splenocytes in TCM. The titrated vAPCs were then mixed with an equal volume of responder splenocytes from either immunized or naïve control mice at 10^7^ cells/mL, and 100 μL of APC:responder cell suspension added to wells of pre-coated ELISPOT plates, or flat-bottomed 96 well plates for intracellular cytokine staining (ICS). Plates were incubated for 18 h before processing for cytokine detection by ELISpot, ICS, or supernatant assay by LEGENDplex.

### Intracellular cytokine staining (ICS)

Intracellular cytokine staining was performed using reagents according to the manufacturer’s instructions (Biolegend Inc.). Briefly, cells were harvested from recall assays in round-bottom 96-well plates by centrifuging at 400 × *g* for 5 min, and surface-labeling the cells with an antibody cocktail comprising BV510-anti-CD8, BV650-anti-CD19, BV785-anti-CD4 and 7AAD for 20 min at RT. After washing in Dulbecco’s PBS (DPBS), cells were fixed and permeabilized for 20 min at RT in Cyto-Fast Fix/Perm buffer. After washing 2× in Cyto-FastPerm Wash solution, cells were incubated with an intracellular antibody cocktail comprising PacBlue-anti-Tbet, AF488-anti-IFNγ, PE-anti-Gata3, PECy7-anti-IL4, AF647-anti-IL17A and APCCy7-anti-IL2 in 50 μL per well of Cyto-Fast Perm Wash for 30 min at RT in the dark. After washing with 200 μL of Cyto-FastPerm Wash solution, cells were washed 2 × in Cell Staining Buffer (BioLegend Cat. No. 420201) and 100 μL samples were acquired from 96-well plates on a Novocyte 3000 for 50 s, with FSC-H threshold set at 100,000, and voltage settings such that unstained cells have autofluorescence values between 10^2^ and 10^3^ units. Unstained and single-color stained controls were used to set up the compensation matrix. Flowjo software (BD Inc.) was used for flow cytometry compensation, additional data processing, gating, analysis and rendering figures. Briefly, live lymphocytes were gated by appropriate forward vs side scattering, followed by singlet gating by area vs. height of forward scattering pulse, and followed by 7AAD-gating of live (non-apoptotic and non-necrotic cells). Finally, analysis of each T cell subpopulation was conducted as summarized in the “Results” section.

### B cell labelling

HA proteins (Sinobiological Inc.) were conjugated to fluorescent dyes using the DyLight Microscale Antibody Labeling Kit (Thermo Fisher Scientific, Waltham, MA; Cat. No. 22858) according to the manufacturer’s instructions, as described previously^27^. Briefly, lyophilized HAs were reconstituted in 100 μL H_2_O (1 mg/mL) and 8 μL of 0.67 M borate buffer added. H1, H5 and H7 were added to DyLight405, 488 and 650 (Cat. Nos. 53021, 53025, 8543), respectively, vortexed gently and incubated at RT for 1 h. Excess unbound dye was removed by gentle mixing with 108 μL of Dye Removal Resin (centrifuged prior to mixing to remove the storage buffer) followed by centrifugation at 1000 × *g* to collect the labelled proteins. For B cell labelling, splenocyte cell suspensions were first surface labelled at RT for 30 min with a cocktail of fluorescent Abs and reagents, comprising BV510-anti-CD138, BV655-anti-CD3, BV785-anti-CD19, PE-anti-IgG2a, PE/Cy-anti-GL7, and APC/Cy7-anti-IgM (BioLegend Inc., San Diego, CA) and 7- aminoactinomycin D (7AAD; Thermo Fisher) at 2 × 10^6^ cells/well in 96-well round-bottom plates. After washing in PBS, cells were fixed, permeabilized and washed using an intracellular staining kit (Cyto-Fast from BioLegend) according to the manufacturer’s instructions. Cells were then incubated in a concentration of 2 μg/mL, which is equivalent to 100 ng per well (50 μL total vol) of fluorescently-labeled HA monomers in Cyto-Fast Perm/Wash buffer for 1 h then washed with 200 μL of Perm/Wash buffer. This concentration of fluorescent HA (100 ng per well) was determined to be optimal in titration experiments by having virtually no background staining of plasma cells from nonimmunized mice, and showing good separation of the HA^+^ from HA^-^ population. Data were acquired using a Novocyte 3000 flow cytometer for 50 s, with FSC-H threshold set at 100,000, and voltage settings such that unstained cells have autofluorescence values between 10^2^ and 10^3^ units. Unstained and single-color stained controls were used to set up the compensation matrix. Data were analyzed with FlowJo software. CD19+ (including dim or lo and bright or high) cells were gated in a two-dimensional CD19 vs. CD3 plot, followed by gating of antigen-specific (plasma) cells in a 2D CD138 vs. CD19 plot (plasma cells are defined CD138 + CD19lo). For additional 2D plots, gated plasma cells were plotted in 2D showing H1 vs. H5, H1 vs. H7 and H5 vs. H7 to measure potential cross-reactivity. Additional statistics were carried out using tables and layouts in FlowJo followed by rendering graphs in Excel (Microsoft Corp., Redmond, WA).

### Single cell transcriptomic sequencing and analysis

Three C57Bl/6 female mice were administered 5 μg H5 monomer (Sinobiological Cat. 11062-V08H1) in 50 μL adjuvant (IVAX-1) comprising an equal mixture of AddaVax emulsion (Invivogen) and PBS containing MPLA in liposomes and CpG ODN 1018, at final concentrations of 3 and 1 nmole, respectively. The use of monomers was based on array profiling data comparing responses to H5 trimers and monomers (Supplementary Fig. S1). These profiles were highly correlated when either form of H5 was administered in IVAX-1 via the s.c. route. The formulation was administered the via the s.c. route (base of tail) and boosted similarly on d14, and again on d28 (second boost via i.p. route), and spleen (SP) and lymph nodes (LN) from multiple sites harvested 7 days later. Single cell suspensions of LN cells and erythrocyte-depleted SP were prepared by gently pressing tissues through a cell strainer. SP and LN cell suspensions from two age-matched naïve mice were also prepared for comparison. The four samples (unimmunized LN, unimmunized SP, immunized LN, immunized SP) were then bar coded separately using cell multiplexing kits (3' CellPlex Set A, 10X Genomics, Cat. No. 1000261) at 2 × 10^6^ total cells per sample following manufacturer’s protocol. The final barcoded cells were resuspended at ~ 2000 cell/μL, and then mixed to form two pools: one comprised unimmunized LN and SP cells (~ 5000 each) in one droplet encapsulation and cDNA library preparation channel, and the other immunized LN and SP (~ 5,000 cells each) similarly prepared in another channel. These two channels were subjected to single-cell cDNA library preparation (10× Genomics Chromium 3’ v3 kit) and then sequenced with a target depth of ~ 50 K reads per cell using the Illumina HiSeq system. For read alignment and data preprocessing, FastQC^30^ was first used to quality control FastQ format raw data using the fastqc command and the –noextract flag. The multiplexed samples were then demultiplexed, and the FastQs were aligned to the CellRanger^31^ reference mm10-3.0.0 using STAR implemented in CellRanger v6.0.2 with the multi command and default parameters. The output .mtx and feature barcode files from CellRanger were read into SCANPY v1.7.2^32^. Only the SP data were considered for downstream analyses presented here. The following operations were performed using SCANPY’s built-in functions unless otherwise stated. The resulting count matrices were concatenated such that only genes that were expressed in both unimmunized and immunized mice (hereafter referred to as “batches”) were included. Only cells that were confidently demultiplexed, that were expressing more than 200 genes, and genes that were expressed in more than 3 cells, were considered for the downstream analysis. The count data were log-normalized using the “Seurat” method^33,34^. For data batch-correction, the data were reduced to 50 dimensions using principal component analysis. The principal components were batch-corrected using harmonypy implemented in SCANPY with defaults. The k-nearest neighbor graph was constructed on the batch-corrected principal components with 100 neighbors and cosine distance metric. The resulting graph was reduced to 2 dimensions using UMAP for visualization. The cell types were identified using a support vector machine (SVM) model implemented in scikit-learn v0.24.2^35^ with a sigmoid kernel and C = 10 trained on unpublished single cell transcriptomic data. The features (genes) were subset to those that were present in both datasets, the features in the training dataset were fit and scaled using scikit-learn’s StandardScaler function, and the features in the splenocyte dataset were scaled with the same scaler; 20% of the training data were held out for validation. Genes that were differentially expressed between different cell types were identified using the Mann–Whitney-U rank-sum test, with Benjamini–Hochberg correction^36^. Ribosomal genes (Rps and Rpl) were excluded from the testing. Only genes with an absolute value of log_2_ fold change > 0.5 were considered. Scrublet^37^ v0.2.3 was used to determine doublet probabilities. Scrublet pairs random transcriptomes together to form a training dataset, which it then uses to train a k-nearest neighbors doublet classifier. The scrublet pipeline was run as specified by the documentation with defaults.

Cell–cell communication inferences were made using CellChat v1.1.2, a recently developed tool that generates and plots cell–cell communication probabilities and interaction strengths from single cell transcriptomic data^38^. The normalized data were exported from SCANPY and imported into Seurat v4.0.3 using custom functions. The CellChat interaction strengths were generated for each dataset individually (with min.cells = 100, all other parameters were left as defaults) and then combined. The differential interaction strength was plotted using the netVisual_diffInteraction function in CellChat with defaults. The comparison between all unvaccinated and vaccinated cells was performed by subsetting the treatment naïve and vaccinated groups until each celltype had the same number of cells in each group, then doing differential expression as above.

### Other data analysis and statistics

Protein microarray data were tested for significance between groups using two- tailed Mann–Whitney tests for unpaired data or two-tailed Kruskal–Wallis tests with Dunn’s multiple-comparisons using GraphPad Prism 6.7 (GraphPad, La Jolla, CA, USA). A P value of < 0.05 was considered statistically significant. Flow data were analyzed with FlowJo software (FlowJo Llc., Ashland, OR). To determine antibody breadth, geometric means of Log2-transformed data for each antigen were determined for each vaccine group, and an antigen was scored seropositive if above a cutoff defined as the mean + 2SD of the signals for an irrelevant protein on the array (NA) which was not used to immunize. Array data were tested for significance using Kruskal Wallis Dunn's multiple comparison test in R or Prism. Graphical outputs were generated in Prism and Microsoft Excel.

## Results

### Breadth and magnitude of IgG response is influenced by TLR agonist(s) and emulsion

The protein microarray was used to determine the effect of different adjuvant formulations on the breadth of the IgG response. We utilized HA monomers expressed in eukaryotic cells as detection antigens on the array. Therefore, conformation-dependent epitopes present only on the quaternary structure of assembled trimers will not be displayed. Similarly, other conformation-dependent epitopes may be under-represented on the monomers, particularly the HA1 fragment, according to the conformation adopted by the antigen. Nevertheless, the array revealed a hierarchy of responses (characterized by breadth and magnitude) induced by different adjuvants. Groups of C57Bl/6 female mice (7–10 weeks old) were administered via the s.c. route (base of tail) a single dose of trimerized VN04 H5 in a different TLR agonist (TLR a), or combination TLR a, and formulated without or with squalene oil-in-water emulsion (AddaVax) as shown in Table 1. Agonists comprised Pam2CSK4 (TLR2/6 a), pyrimido-indole derivative and monophosphoryl lipid A (both TLR4 a), imidazoquinoline derivative (TLR7 a), ODN 1018 CpG DNA containing a phosphorothioate backbone (TLR9 a), and a chemically linked tri-agonist (TLR2/6_4_7) as described previously^25^. The pyrimido-indole TLR4 a is less potent than the conventional MPLA agonist for TLR4, but is more suited for tri-agonist synthesis^25,39^. Plasma samples were obtained at days 10, 28, 43 and 103 for probing against an influenza protein microarray described previously^27^, which displayed 125 different monomeric full length HA (HA0) drift variants and 131 HA1 variants, spanning all 18 HA subtypes. Other than transient weight loss in the first 24 h after vaccination, the animals in each group showed normal increases in body weight (Supplementary Fig. S2).

**Figure 1:**
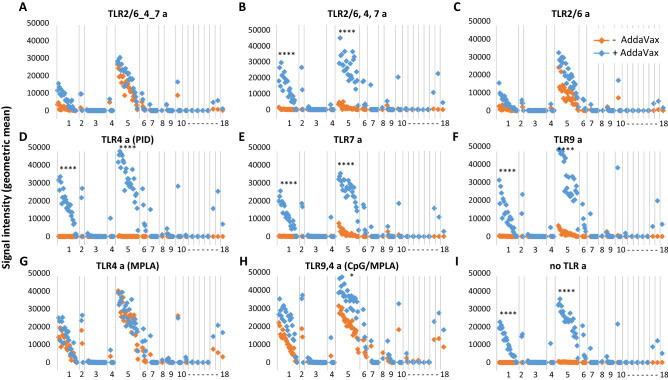
IgG profiles in response to trimerized influenza virus VN04 H5 vaccine formulated with different TLR agonists, with or without squalene oil-in-water emulsion (AddaVax). Antibody profiles were determined by probing immune plasma (d28-post vaccination) against HA protein microarray (Supplementary Table S1). Antigens (horizontal axis) are full-length (HA0) HA variants ranked within subtype H1-H18 by overall average of whole study population. Magnitude of response (vertical axis) are geometric means of each vaccine group (N = 4 mice). Breadth of response is summarized in Table 2. *CpG* cytosine-guanine oligo deoxynucleotide, AddaVax; *MPLA* monophosphoryl lipid A; *PID* pyrimido-indole derivative; *TLRa* TLR agonist. Kruskal Wallis Dunn's multiple comparison between + and − AddaVax are indicated for H1 and H5; ****P < 0.0001; ***P ≤ 0.001; **P ≤ 0.01; *P < 0.05.

**Figure 2:**
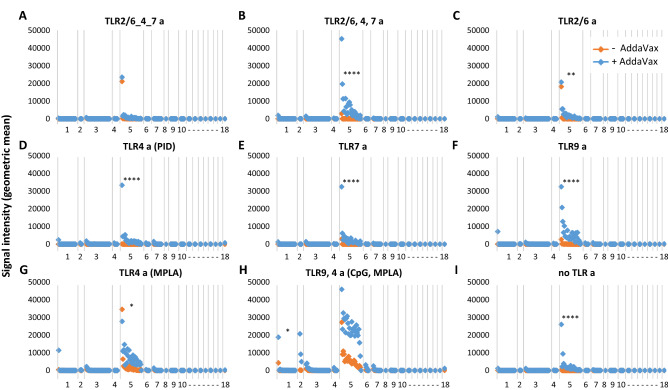
IgG profiles in response to trimerized influenza virus VN04 H5 vaccine formulated with different TLR agonists, with or without squalene oil-in-water emulsion (AddaVax). As per Fig. 1, except arrayed antigens (horizontal axis) are HA1 regions of HA. Abbreviations, as Fig. 1.

IgG responses peaked ~ d28–d43; the IgG profiles by protein microarray for full-length HA0 and HA1 on d28 are shown in Figs. 1 and 2, respectively. Full-length H5 drift variants on the array range overall from 90.3 to 99.8 (average 96.4) % sequence identity with the immunizing HA (VN04 H5), while drift variants of H1 (the closest phylogenetic group to H5) range from 59.5 to 65.6 (average 62.9) % sequence identity with VN04 H5. These values are higher if the conserved HA2 regions are compared. Thus, 92.9–99.6 (average 97.8) % identity for H5 variants, and 75.7–77.9 (average 77.1) percent identity for H1 variants. In addition to H1, H5 immunization also induces heterosubtypic cross-reactivity for other closely-related Group 1 HAs, including H2 and H6. In the absence of AddaVax emulsion (orange symbols in Figs. 1 and 2), the magnitude of responses were greatest with MPLA and the CpG/MPLA combination adjuvant (Fig. 1G,H). The linked TLR 2/6_4_7 tri-agonist and TLR2/6 a also induced robust H5 responses (Fig. 1A,C), while unlinked TR2/6,4,7 tri-agonist, TLR7 a, and TLR9 a induced weak, but measurable homosubtypic IgG cross-reactivity only without emulsion (Fig. 1B,E,F, respectively). H5 with pyrimido-indole derivative (TLR4 a), or administered alone without TLR a, failed to induce detectable IgG under the conditions used (Fig. 1D,I, respectively). Comparisons between the different TLR agonists administered without emulsion revealed the magnitude of the response induce by TLR4a (MPLA) and TLR9 a/TLR4 a (CpG/MPLA) was significantly higher than most of the other vaccine groups (Supplementary Table S1). Linked TLR 2/6_4_7 triagonist without emulsion was also significantly higher than TLR4 a (PID) and antigen alone.

AddaVax alone was an excellent adjuvant for H5 (Fig. 1I), and consistently elevated the responses induced by different TLR agonists. Indeed, when magnitudes were compared between emulsified adjuvants, only TLR9a/TLR4a (CpG/MPLA) + AddaVax reached significance over any other groups (Supplementary Table S1). Of particular note, the pyrimido-indole derivative (TLR4 a), which failed to elicit detectable IgG against H5, produced robust homo- and hetero-subtypic cross-reactivity when co-administered with AddaVax. This was higher than achieved with AddaVax or TLR4 a alone, indicative of a synergistic response. AddaVaxThe effect of the emulsion was often dramatic. For example, the cross-reactive response to antigen administered with unlinked tri-agonist, TLR7 a, or TLR9 a, were modest and homosubtypic (H5) only, but homo- (H5) responses and hetero- (H1) responses were significantly elevated when co-administered in AddaVax. Interestingly, the smallest change by inclusion of AddaVax was seen with MPLA (a TLR4 a), which is by itself a strong adjuvant for H5; the profile after coadministration with AddaVax was essentially unchanged. However, this may be related to the liposome needed for delivery of hydrophobic MPLA which may not benefit from an oil-in-water emulsion in the same way as other water-soluble agonists.

The breadth induced by each formulation is summarized in Table 2. In the absence of AddaVax, all formulations (with the exception of the pyrimido-indole derivative), induced broad homosubtypic responses to H5 variants, with TLR4a (MPLA) and TLR9 a/TLR4 a (CpG/MPLA) inducing the broadest. Heterosubtypic cross-reactivity was more restricted. For example, cross-reactivity for over half of the H1 variants was induced by MPLA and CpG/MPLA only, with breadth for less than half of H1 variants induced by the linked and unlinked triagonist and TLR 2/6 a. TLR4 (pyrimido-indole derivative), TLR7 a and TLR9 a failed to induce cross-reactivity to H1 or any other Group 1 HAs. Cross-reactivity was also generated against H6 by four of the nine non-emulsion TLR a formulations, with CpG/MPLA inducing the broadest response of the four. CpG/MPLA was also the only non-emulsion formulation to induce cross-reactivity to the more phylogenetically distant H8 (100%) and H11 (33%). In contrast, all formulations containing AddaVax, including AddaVax alone, induced a maximally broad response (100%) against H5 and H6, and near maximal responses against H1 and H2.

**Table 2:**
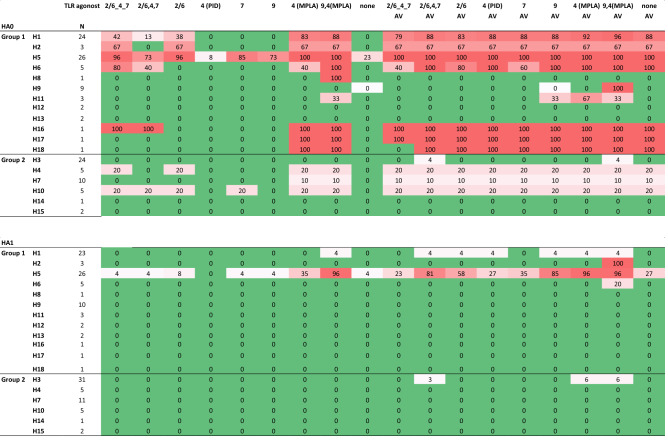
Summary of breadth of response elicited by H5 administered in different adjuvants.

Values are percentages of seropositive variants within each HA subtype printed on the array (both full-length HA0 and HA1 fragment) using data shown in Figs. 1 and 2. HA subtypes are organized into phylogenetic group 1 or 2. The cutoff for seropositivity was defined for each formulation with Log2 transformed data using the mean + 2SD of an irrelevant antigen, neuraminidase.

*N* number of variants in each subtype on the array, *AV* AddaVax, other abbreviations as per Fig. 1.

HA1 fragments contain the variable head domain and part of the more conserved stalk of the protein. Previous studies with this array format have shown that sera with reactivity to the full-length (HA0) may also recognize HA1, depending on whether there are antibodies present against the head^27,40^. IgG profiles to HA1 fragments are shown in Fig. 2 with breadth of response shown in Table 2. The TLR9 a/TLR4 a (CpG/MPLA) combination adjuvant induced the broadest IgG homosubtypic cross-reactivity for HA1 fragments of H5 in the absence of emulsion (Fig. 2H); the magnitude of this was elevated by combining with AddaVax although this increase was not significant. Responses were also induced by other formulations. Several of these were significantly higher when administered in AddaVax compared to without (Fig. 2B–F,I), although these were characterized by reduced breadth (Table 2) and magnitude (Fig. 2) compared to emulsified CpG/MPLA. The weaker response to HA1 compared to full length HA0 may reflect the smaller size of the HA1 polypeptide, and consequently the fewer B cell epitopes available for recognition by the polyclonal response. Alternatively, the conformation of HA1 may not accurately reflect the conformation of the same sequence within the trimerized full-length protein, which may also contribute to the reduced strength of the response. To this point, we presented data previously^27^ showing that conformation-sensitive mAbs raised against H1 from the 2009 pandemic recognized both HA0 and HA1 on the array, suggesting the HA1 is folded correctly.

Hetero-subtypic cross-reactivity against HA1 fragment was minimal. Several of the emulsified adjuvants induced a limited response to a single H1 variant HA1 (4% breadth) while only the emulsified CpG/MPLA adjuvant also induced a response to all three of the H2 variants displayed (Fig. 2H).

**Figure 3:**
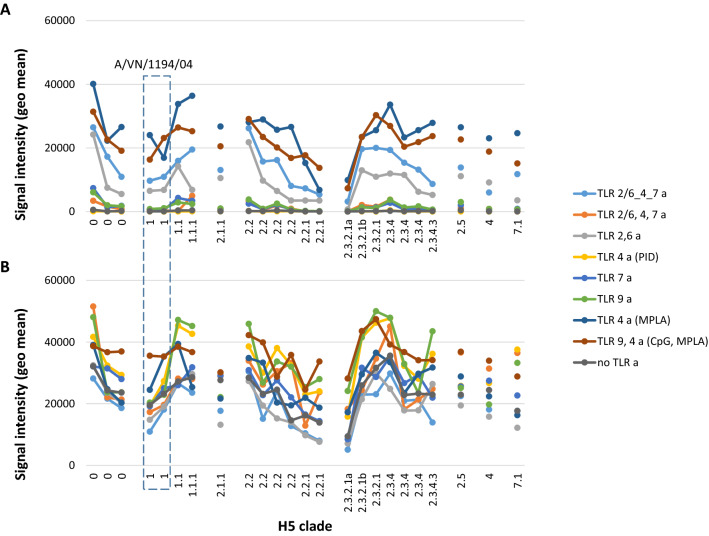
Cross-clade homosubtypic cross-reactivity measured by protein microarray. IgG profiles in response to trimerized influenza virus VN04 (A/VN/1203/2004) H5 vaccine formulated with different TLR agonists, with or without squalene oil-in-water emulsion (AddaVax). H5 antigens (horizontal axis) are full-length (HA0) HA variants that have been organized by clade (classifications from www.fludb.org). Signal intensities (vertical axis) are geometric means of each vaccine group (N = 4 mice). (**A**) minus AddaVax; (**B**) with AddaVax. Boxed, two VN04 H5 proteins on the array (expressed in human or insect cells) that correspond with immunizing H5 variant. Abbreviations, as Fig. 1.

H5 has undergone antigenic drift in poultry and natural wildfowl populations, and different H5 variants are typically grouped into clades and subclades^41^. A successful pre-pandemic vaccine will need to provide broad coverage across different clades to provide anticipatory protection. Thus, Fig. 3 shows array data of H5 variants by clade, which reveals broad cross-clade reactivity generated by the majority of adjuvants tested. In the absence of AddaVax, cross-clade reactivity is the strongest in TLR9 a/TLR4 a, (CpG/MPLA), TLR 4a (MPLA), linked TLR 2/6, 4_7 a, and TLR 2/6 a groups. In the presence of reactivity is elevated for all adjuvant groups.

**Figure 4:**
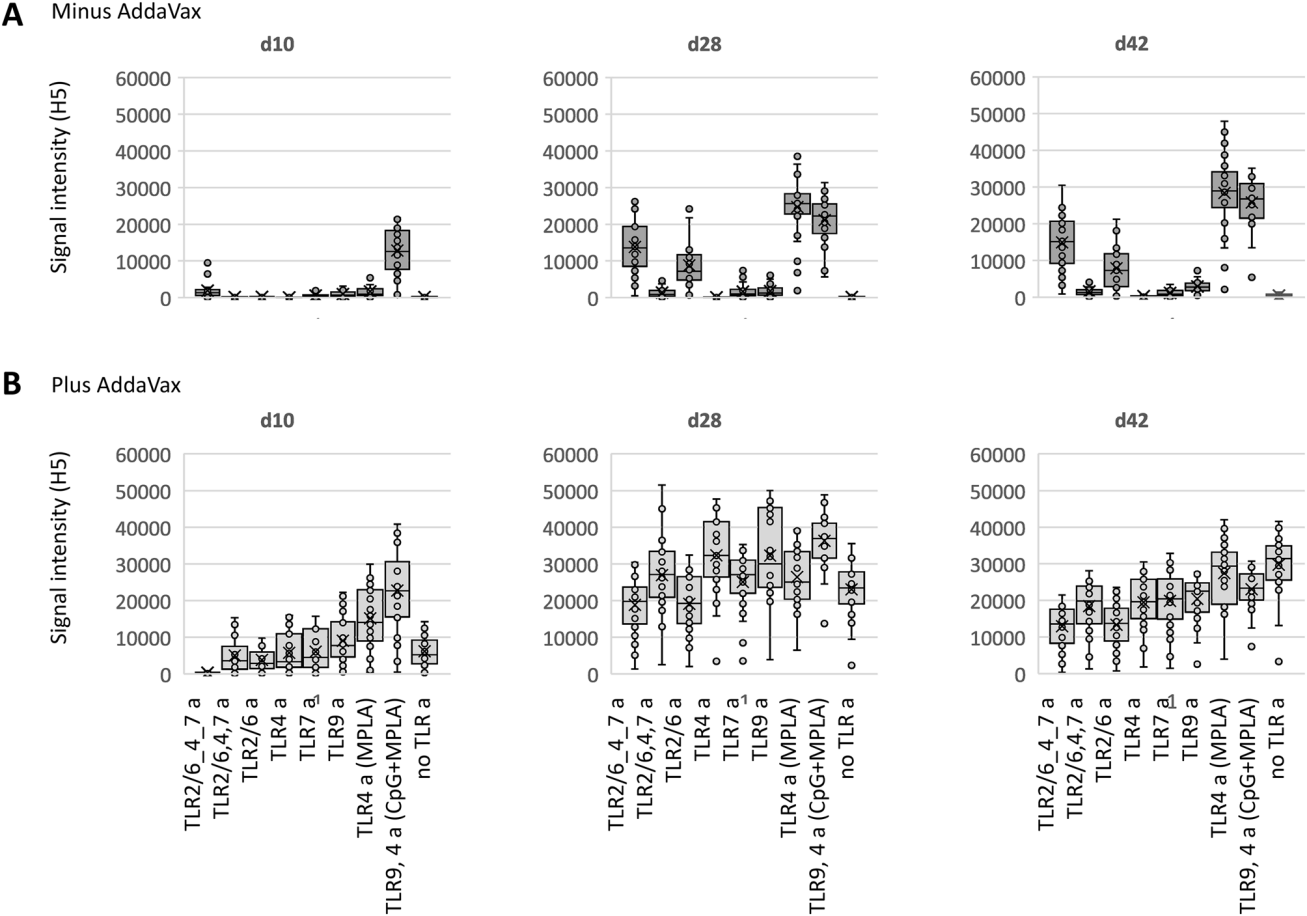
Dynamics of IgG profiles in response to trimerized influenza virus VN04 H5 vaccine formulated with different TLR agonists. Box plots of H5 drift variants; each spot corresponds a different H5 variant on the microarray (geometric mean of 4 mice). Different TLR agonists, listed at the bottom, were administered (**A**) without or (**B**) with squalene oil-in-water emulsion (AddaVax). Abbreviations, as Fig. 1.

In addition to antibody profiles, we assessed the dynamics of the response. Figure 4 shows the development of homosubtypic reactivity at 3 time points after a single dose. In the absence of AddaVax, the TLR9 a/TLR4 a (CpG/MPLA) combination adjuvant produced the most rapid response, being the only vaccine to have elicited detectable antibodies on day 10. By day 28, IgG reactivity was also seen from linked TLR 2/6_4_7 a tri-agonist, TLR2/6 a, and TLR4 a (MPLA), which remained elevated at day 42. In the presence of AddaVax, all the vaccines elicited reactivity by day 28, although CpG/MPLA/AddaVax again elicited the most rapid response, as can be seen on day 10. We conclude from these data, and data presented in Figs. 1 and 2, that of all the adjuvants tested, CpG/MPLA/AddaVax induces a broad response that appeared more quickly and reached a greater magnitude than the other formulations tested. The response was also durable after a single dose, remaining high at d42. Of interest, at d42 the responses induced by AddaVax alone or TLR4 a(MPLA)/AddaVax remained high, and indeed higher than CpG/MPLA/AddaVax.

### Th1/Th2 balance is variable according to adjuvant used

**Figure 5:**
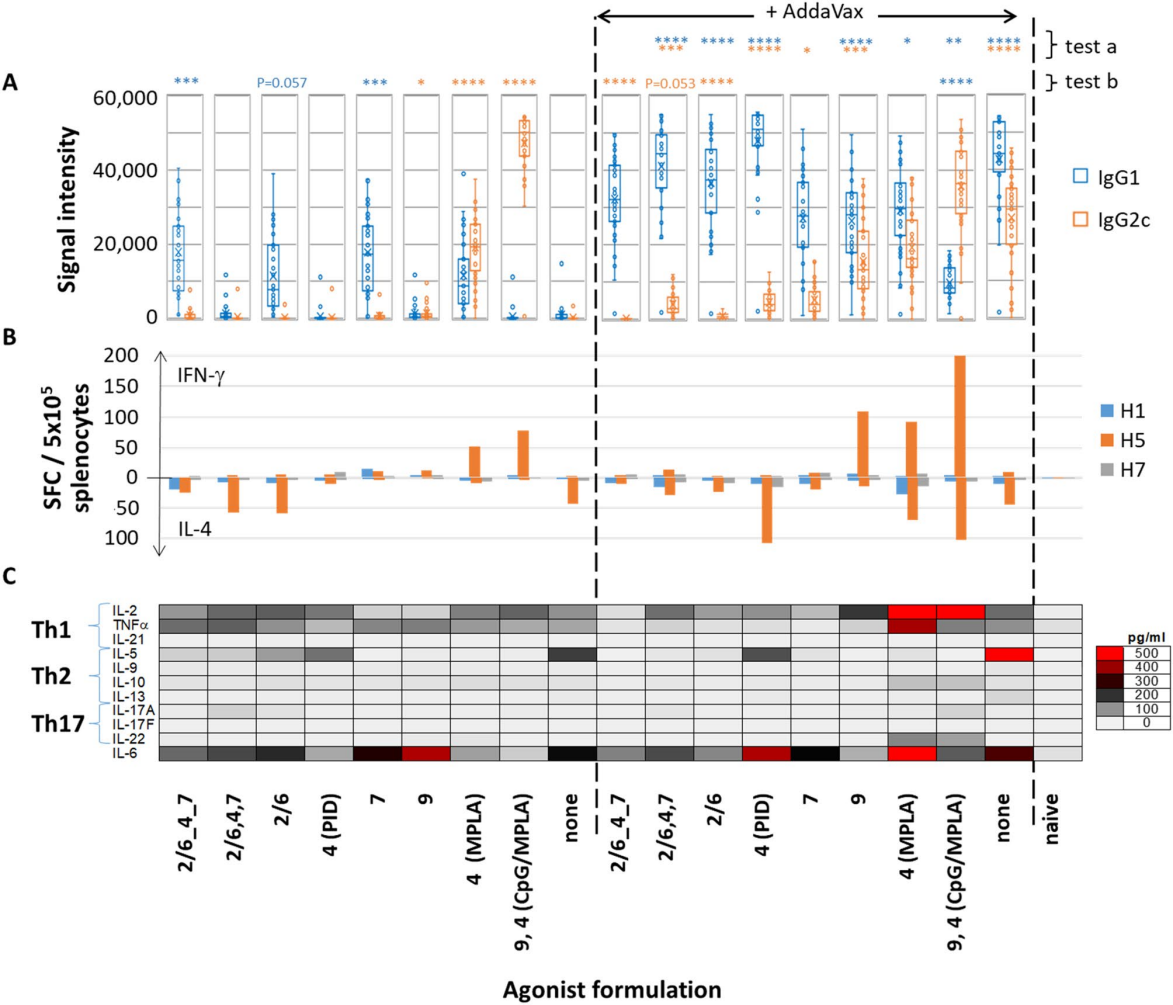
Th1 vs. Th2 balance in response to H5 vaccine formulated with different TLR agonists, with or without AddaVax. (**A**), Box plots of IgG1 and IgG2c profiles for H5 drift variants; each spot corresponds a different H5 variant on the microarray (geometric mean of 4 mice). Kruskal Wallis Dunn's multiple comparison tests are shown above each column; test a, + AddaVax vs. − AddaVax; test b, left side, each TLR a/− AddaVax vs. no TLR a/− AddaVax; right side, each TLR a/+ AddaVax vs. no TLR a/AddaVax; ****P < 0.0001; ***P ≤ 0.001; **P ≤ 0.01; *P < 0.05 (colors correspond to IgG isotype). (**B**) Spot-forming cells (SFC) in IFN-γ and IL-4 ELISpots performed on the same mice as shown in (**A**) 10 days post i.p. boost on d56 (means of 4 mice), using H1, H5 and H7 as recall antigens. Cell viability confirmed using Con-A mitogen (not shown). (**C**) Multiplex cytokine bead array (LEGENDplex) of culture supernatants of ELISpots shown in (**B**). Other abbreviations, as Fig. 1.

Both IgG1 v IgG2c subtyping on microarrays and cytokine profiling in T cell recall assays were performed on the same groups of immunized C57Bl/6 mice to evaluate the Th1/Th2 response elicited by each adjuvant tested (Fig. 5). In the absence of AddaVax emulsion, TLR2/6_4_7 a tri-agonist, and individual TLR2/6 a and TLR7 a stimulated IgG1 antibodies (Th2) (Fig. 5A). In contrast, TLR9 a/TLR4 a (CpG/MPLA) was strongly polarized to IgG2c (Th1), while TLR4 a (MPLA) gave a more balanced response. AddaVax was an excellent adjuvant in its own right, significantly elevating both IgG1 and IgG2c compared to H5 without adjuvant. When combined with TLR agonists, AddaVax elevated IgG1, with all but two (linked triagonist and TLR7 a) reaching significance (test a in Fig. 5A). AddaVax also significantly elevated IgG2c from four of the TLR agonists (test a); interestingly, several were lower than the IgG2c response achieved by AddaVax alone (test b). AddaVax also significantly elevated IgG1 from TLR9a/TLR4a although the response was still IgG2c/Th1 polarized. Responses to TLR9 a (CpG) and TLR4 a (MPLA) were more balanced with both IgG1 and IgG2c detected. It is noteworthy that in the absence of AddaVax responses to some TLR agonists were undetectable (i.e., unlinked tri-agonist, and individual TLR4 (PID) and TLR9 agonists); however, in the presence of AddaVax all three were strongly immunogenic.

IFN-γ and IL-4 release was determined from the same mice at the experimental endpoint by ELISpots (Fig. 5B). The recall assay included immunizing (H5) antigen, as well as hemagglutinins H1 and H7 (63% and 42% sequence identity with H5, respectively). Despite observing heterosubtypic cross-reactivity at the Ab level, IFN-γ and IL-4 spot-forming cells (SFCs) were detected against H5 only. Several cytokine patterns were consistent with IgG1/IgG2c profiles. For example, in the absence of AddaVax, the only two groups to induce IgG2c were agonists to TLR4 (MPLA) and the combination TLR9 a/TLR4 a (CpG/MPLA) adjuvant; accordingly, these also produced H5-specific IFN-γ SFCs in the recall assay. In the presence of AddaVax, these two agonists elicited elevated signals to IgG1 which correlated with the presence of IL-4 SFCs. Similarly, in the absence of AddaVax, linked TLR 2/6_4_7 tri-agonist and TLR2/6 and 7 agonists produced only IgG1 (Th2) and no IFN-γ SFCs, of which two (linked TLR tri-agonist and TLR2/6 a) produced detectable IL-4 SFCs. In the presence of AddaVax, linked and unlinked TLR tri-agonist, and individual TLR 2/6, 4 and 7 agonists produced strongly polarized IgG1 (Th2) responses and correspondingly low numbers of IFN-γ SFCs but detectable IL-4. This contrasts with AddaVax alone, which produces a balanced IgG1/IgG2c profile, suggesting these TLR agonists suppress the production of IgG2c. Of note is the TLR4a, PID, which appears ineffective at inducing antibody or cytokines in the absence of AddaVax, but which becomes a strong Th2-polarizing adjuvant in the presence of AddaVax, displaying strong IgG1 signals and robust IL-4 SFC numbers. Note this contrasts with the combination TLR9 a/TLR4 a (CpG/MPLA) in the absence of emulsion, which strongly polarizes the response to IgG2c (Th1) and a production of IFN-γ SFCs. Thus, the breadth of the cross-reactive response and polarization can be tuned according to the adjuvant, as summarized in Table 3.

**Table 3:** Summary of the effect of different adjuvants on homo- (H5) and hetero- (H1) subtypic cross-reactivity and polarization of the Th1 vs Th2 response.

Agonist	Without AddaVax				With AddaVax			
	IgG H5	IgG H1	IgG1 IL-4	IgG2c IFN-γ	IgG H5	IgG H1	IgG1 IL-4	IgG2c IFN-γ
TLR2/6_4_7	++	−	++ ++	− −	++	+	++++ +	− −
TLR2/6, 4, 7	−	−	− +++	− −	+++	++	+++++ ++	+ +
TLR2/6	+	−	+ +++	− −	++	+	++++ +	− −
TLR4 (PID)	−	−	− +	− −	++++	++	+++++ ++++	+ −
TLR7	−	−	++ −	− +	+++	++	+++ +	+ −
TLR9	−	−	− −	− +	++++	++	+++ +	++ ++++
TLR 4 (MPLA)	+++	+	+ −	++ +++	+++	++	+++ ++	++ ++++
TLR 9, 4 (CpG, MPLA)	+++	++	− −	+++++ ++++	++++	+++	+ ++++	++++ ++++
None	−	−	− ++	− −	+++	++	++++ ++	+++ +

Data for homo- and heterosubtypic cross-reactivity from Fig. 1, data for Th1 and Th2 response from Fig. 3. Key: For IgG, IgG1 and IgG2c, median signal intensities −, < 2000; +, 2000–9999; ++, 10,000–19,999; +++, 20,000–29,999; ++++, 30,000–39,999; +++++, > 40,000. For numbers of IL-4 and IFN-γ spot forming cells (SFCs) per 5 × 10^5^ splenocytes: −, < 10; +, 10–24; ++, 25–49; +++, 50–75; ++++, > 75.

The largest numbers of IFN-γ and IL-4 producing SFCs was induced by the combination of MPLA, CpG and AddaVax. This appears to result from synergy between AddaVax and either TLR agonist, as the SFC numbers for MPLA + AddaVax or CpG + AddaVax were greater than the sum of SFC numbers for the individual components. Measurements of antigen-specific B cells also suggest these components are synergistic (see “CpG and MPLA synergize in the induction of antigen-specific plasma cells” below).

Additional cytokines were assayed in the supernatants of recall assay using multiplex bead detection (Fig. 5C). The highest concentrations were elicited by MPLA/AddaVax and CpG/MPLA/AddaVax combination adjuvants. The former produced high levels of Th1-associated IL-2 and TNF-α, as well as high levels of IL-6, which is produced by different APCs and promotes Th2 differentiation^42^. These data are consistent with the balanced IgG1/IgG2c profile seen by arrays and ELISPOTs. CpG/MPLA/AddaVax also produced high levels of IL-2 and TNF-α, but lower levels of IL-6, which may contribute to the skewing toward IgG2c over IgG1. Both MPLA/AddaVax and CpG/MPLA/AddaVax also induced modest levels of Th17-associated cytokines. Among the non-emulsified formulations (left side of Fig. 5), of note is CpG/MPLA. This adjuvant is strongly biased to Th1 with elevated IL-2 and TNF-α but low IL-5 and IL-6, consistent with the strongly polarized IgG2c response. MPLA produces a more balanced Th1/Th2 cytokine profile, consistent with the balanced IgG1/IgG2c response. The remaining non-emulsified agonists, which produced mainly IgG1 skewed responses, produced both Th1 and Th2-associated cytokines. Interestingly, H5 alone (without TLR agonist or emulsion), which did not induce detectable antibodies, was associated with the release of several cytokines in the recall assay. This profile was amplified by AddaVax, notably release of IL-5. This would suggest H5 has an inherent capacity to stimulate a T cell response, as has been reported by others^43,44^, although the mechanism is unknown.

### CpG and MPLA synergize in the induction of antigen-specific plasma cells

**Figure 6:**
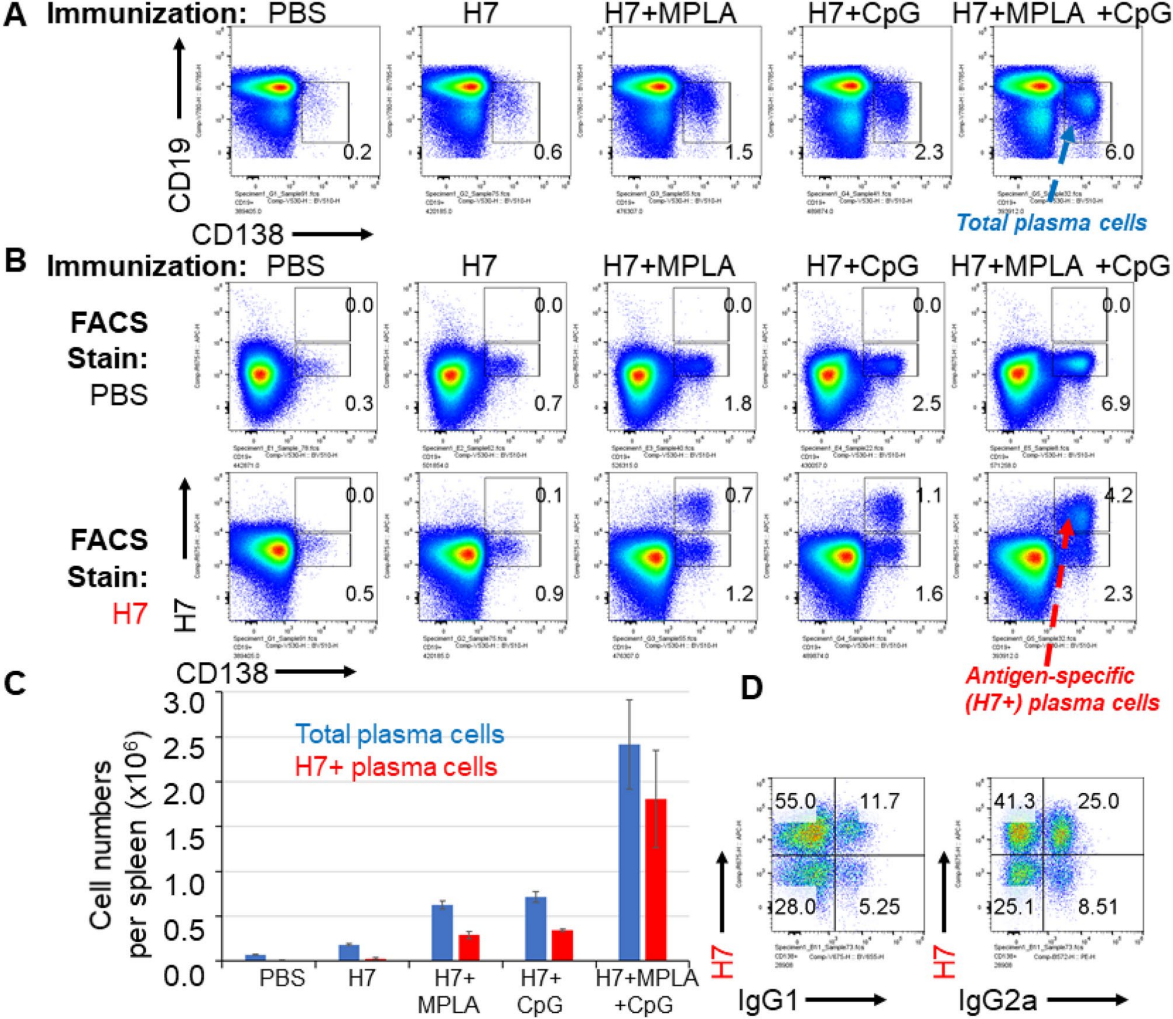
MPLA and CpG synergize to induce HA-specific plasma cell formation in vivo. Mice were immunized on day 0 with PBS (control), or influenza virus HA (H7 from A/Anhui/1/2013) alone or with MPLA, CpG or MPLA/CpG, all in the presence of AddaVax. Mice were boosted i.p. on day 14, and spleens harvested on day 19. (**A**) Plasma cells, defined as CD138^+^, CD19lo; (**B**) antigen-specific plasma cells; (**C**) numbers of total (blue) and antigen-specific (red) plasma cells; (**D**) total plasma cells, defined as CD19lo CD138+ as in (**A**) above, are plotted to visualize H7 fluorescence vs IgG1 or IgG2a expression. H7-specific plasma cells induced by immunization with IVAX1 produce predominantly Th1-associated IgG2a.

It was of interest to determine whether the effects of combining CpG and MPLA in AddaVax were synergistic, since synergies may allow lower doses of adjuvant to be used and development of less reactogenic vaccines. To address this, we used antigen-specific B cell labelling to quantify these in mice administered influenza HA in CpG/MPLA/AddaVax, and compared these with CpG/AddaVax or MPLA/AddaVax individually. The CpG/MPLA/AddaVax combination adjuvant induced more total plasma cells (Fig. 6A) and antigen-specific B cells (Fig. 6B) than the sum of CpG/AddaVax and MPLA/AddaVax separately, indicating a synergistic effect. Almost all the antigen-specific B cells were CD138+, indicating their differentiation into plasma cells. These numbers are shown as bar charts in Fig. 6C. IgG isotype staining of antigen-specific plasma cells revealed both IgG1 and IgG2a are produced (Fig. 6D), although the majority expressed IgG2a, consistent with the Th1>Th2 polarization seen by array profiling, and T cell recall assay (Fig. 5).

### Further characterization of the utility of IVAX-1 as an adjuvant

**Figure 7:**
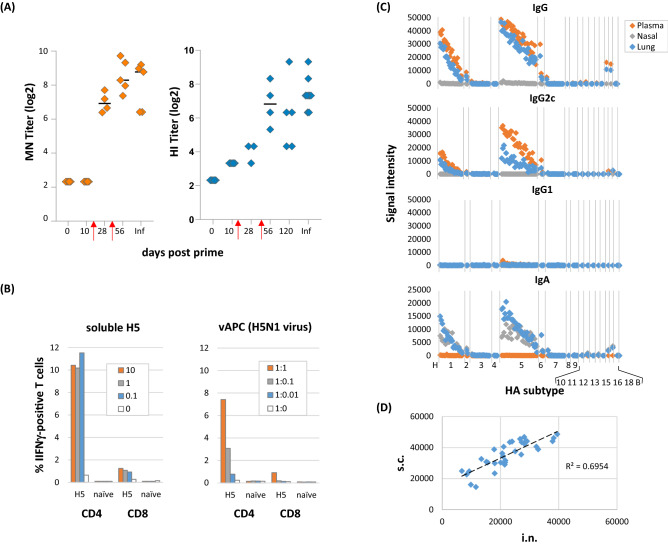
Further characterization of H5/IVAX-1. Mice were administered trimerized H5 antigen from A/Viet Nam/1203/04 in combination adjuvant CpG/MPLA/AddaVax (“IVAX-1”). (**A**) Virus microneutralization (MN) and hemagglutination (HI) assays. Five C57Bl/6 mice were immunized and boosted on days 14 and d42 (red arrows), and plasma samples collected at different time points tested for neutralization against reassortant H5N1 virus. Plasma from H5N1-infected mouse (Inf) was used as a positive control. Neutralizing titers against H5N1 virus increase with boosting. (**B**) IVAX-1 preferentially stimulates CD4^+^ T cells compared to CD8^+^ T cells. T cell recall assays were performed by incubating splenocytes from H5/IVAX1-immunized mice with recall antigens as either soluble H5 protein (monomers from Sinobiological) at different concentrations (mg/mL), or syngeneic vAPCs (splenocytes exposed for 1 h to reassortant H5N1 influenza virus) at different splenocyte : vAPC ratios. Cultures were incubated overnight, and the proportions of IFNγ-positive CD4 and CD8 cells determined using ICS. (**C**) H5/IVAX-1 administered by intranasal (i.n.) route induces IgG2c in lung and plasma, and IgA in lung and nasal turbinate washes. B6 mice were administered H5 trimers in IVAX-1 adjuvant via the i.n. route on d0 and d56. Antibody profiling was performed on d69 using protein microarrays probed with plasma and lung and nasal washes. Antigens are ranked left-to-right in the same order in each panel indicating the IgG and IgA profiles are essentially identical. (**D**) Administration of H5/IVAX-1 via the i.n. route induces essentially identical profiles to administration via the s.c. route. Shown are scatter plots of Abs signals against different H5 variants on protein microarrays. Plasma samples were obtained after i.n. and s.c. administration (x and y axes, respectively); each point represents a different H5 drift variant.

We then performed a series of experiments to characterize CpG/MPLA/AddaVax combination adjuvant (IVAX-1) further. Since array is a non-functional assay, it was of interest to determine if IVAX-1/H5 induced virus neutralization. Figure 7A shows plasma generated using VN04 H5/IVAX-1 neutralized H5N1 reassortant virus in vitro. We noticed, as reported previously^27^, that boosting was required to reach detectable levels of neutralization. We showed earlier that IVAX-1 engendered a robust IFNγ-response in the ELISpot (Fig. 5B). As the antigen for recall was supplied as soluble H5, it was expected that most of the processed antigen would be presented in the context of class II MHC to CD4 T cells, rather than to CD8 T cells. This was confirmed when intracellular cytokine staining (ICS) was used in place of the ELISpot to identify the source of IFNγ (Fig. 7B). Therefore, we attempted to improve the recall of CD8 T cells using H5N1 influenza virus-infected splenocytes (vAPCs). However, we saw no improvement in detection of antigen-specific CD8 cells. Although the data support the notion that IVAX-1 preferentially stimulates CD4 T cells, we cannot exclude a CD8 T cell response as infected respiratory epithelial cells (the natural host cell for IAV) may be required for vAPCs instead of splenocytes. We also explored intranasal (i.n.) administration of IVAX-1, since the above experiments were based on the s.c. route only. The i.n. route is often used to engender an IgA response in the nasal and respiratory mucosa. Mice were administered H5/IVAX-1 via the i.n. route and boosted on d56. Other than transient weight loss (< 8%) within 24 h of the prime and boost, there were no adverse events associated with this intranasal delivery or boosting (not shown). At the experimental endpoint (d69), IgG, IgG1, IgG2c and IgA profiles were determined for plasma, and nasal and lung washed by microarray (Fig. 7C). Robust IgG responses were seen in plasma, dominated by subtype IgG2c, consistent with the findings seen when the same vaccine was administered by the s.c. route, as shown in Fig. 5. Robust IgA responses were found in nasal and lung washes, and IgG was also found in the lung, although no IgA was detected in plasma. Ab profiles recognized by IgG, IgG2c and IgA were identical after i.n. administration, and comprised recognition of both H5 (homo-subtypic) and H1 (hetero-subtypic) variants. Indeed, the IgG profile induced by i.n. administration was highly correlated with that induced by s.c. administration (Fig. 7D).

### Single-cell mRNA sequencing (sc mRNA seq) analysis

**Figure 8:**
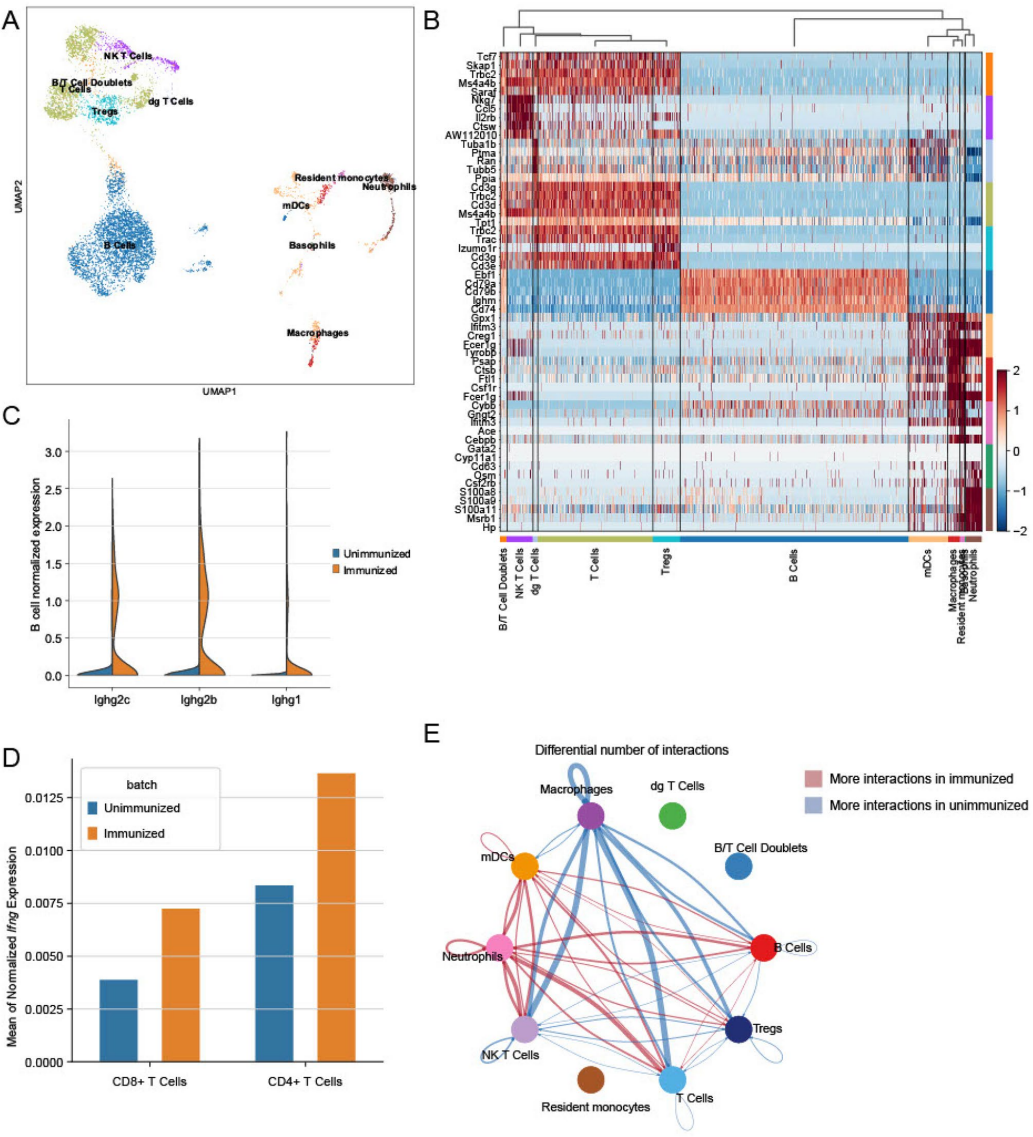
Analysis of single cell transcriptomic data in unimmunized and H5/IVAX-1 immunized mice. C57Bl/6 female mice (N = 3) were administered 5 μg H5 in 50μL IVAX-1 adjuvant s.c. and boosted on d14 (s.c.) and d28 (i.p.), and spleens collected 7 days later. Splenocytes from a single age-matched naïve mouse were also prepared for comparison. (**A**) UMAP visualization of splenocytes before and after vaccination shows transcripts are derived mostly lymphoid lineage with some myeloid cells. (**B**) Heatmap of differential expression testing; the highest differentially expressed genes for most major populations are their marker genes (see text for details). Each column represents a single cell. (**C**) Expression of B cell *Ighg2c, Ighg2b* and *Ighg1* genes is elevated after immunization, with more IgG2 transcripts compared to IgG1. (**D**) Mean expression of *Ifng* gene in CD8+ and CD4+ T cells before and after immunization showing upregulation in both subsets. (**E**) Circular plot showing the main cell–cell interactions in pre- and post-vaccination splenocytes (blue and red lines, respectively).

Finally, in order to generate a transcriptomic “fingerprint” of IVAX-1 for future comparison with other adjuvants, we performed an unbiased single cell transcriptomic analysis of whole spleen cell suspensions pooled from 3 immunized B6 mice that received H5/IVAX-1 barcoded using the 10× Genomics platform and sequenced on the Illumina HiSeq, and performed a similar analysis on an aged-matched naïve control B6 mouse for comparison (see methods). A previously trained supervised support vector machine model (SVM) was first used to define different cell types based on transcriptional profiles. The majority of splenocytes were of lymphoid origin with some myeloid cells, as shown in the Uniform Manifold Approximation and Projection (UMAP) plot in Fig. 8A and Supplemental Fig. S3A. The heatmap in Fig. 8B shows expression profiles of transcriptionally active splenocytes in mice receiving H5/IVAX-1. Cell populations were identified by differential gene expression of the subtype compared to all other subtypes. Overall, three main arms are delineated: T lymphocytes (which also includes NK/T cells, γδ T cells, and Tregs), B lymphocytes, and myeloid cells (myeloid dendritic cells, macrophages, monocytes and neutrophils). Interestingly, the unbiased hierarchical clustering of cell type transcription profiles placed B cells more closely related to myeloid cells than to T cells. The major cell populations were well delineated by marker genes. Thus, the T cells cluster show elevated expression of *Trac, Trbc2, Cd3g and Cd3d* (TCR α and β chain constant domains, and CD3γ and δ chains, respectively). Others include *Ms4a4a* and *b* (CD20 homolog expressed on mature Th1 cells^45^); *Tcf7* (T cell-specific transcription factor^46^); and *Skap1* (regulator of TCR signaling through MAP kinase^47^). *Nkg7* and *Ctsw* genes (NK cell granule protein 7 and cathepsin W) are particularly elevated in NK/T cells and play roles in cell-mediated killing by NK and CD8 T cells^48,49^. Also of note is expression of *Il2rb* (IL2 receptor β chain) which is expressed in NK/T and regulatory T cells (Tregs) at this time point. Also expressed in Tregs is the signature gene *Izumo1r* (*Folr4*; folate receptor)^50,51^. The B cells arm shows elevated expression of *Cd79a* and *Cd79b* (BCR CD79α and β chains); *Ebf1* (B cell-specific transcription factor^52^); *Ighm* (IgM heavy chain); and *Cd74* (MHC class II invariant chain^53^). Cells in the myeloid cell express *Fcer1g* (IgE Fc receptor); *Tyrobp* (an immunoreceptor-associated tyroskine kinase); and *Ifitm3* (IFN-induced transmembrane protein 3), an anti-viral protein which restricts the entry of several viruses into host cells^54^. Among the myeloid cells, neutrophils show expression of S100 family proteins, including *S100a8* and *S100a9* (myeloid-related proteins 8 and 14), which are known to be released by activated phagocytes and amplify the inflammatory effect of LPS mediated through TLR4^55^. Also expressed in neutrophils is *Msrb1* (methionine reductase) which appears to enhance the expression of anti-inflammatory cytokines by macrophages^56^, and *Hp* (haptoglobulin), a major acute phase protein. These data serve as an additional verification that the SVM correctly identified the cell types. Interestingly we found a small population of B and T cell doublets which were identified due to their expression of both T cell markers (Cd3g) and B cell markers (Cd79a) and an unbiased doublet detection algorithm (Supplementary Fig. S3B–D). Doublets in droplet-based sequencing platforms can be indicative of juxtacrine signaling.

We then asked whether the differential IgG2c>IgG1 response characterized above could be corroborated using the transcriptomic data. We found *Ighg2c*, *Ighg2b* and *Ighg1* genes were all upregulated in B cells from immunized mice relative to naïve mice (Supplementary Fig. S4 and Fig. 8C), with log2-fold changes of 6.21 (p-value = 2.14 × 10–93), 4.35 (p-value = 2.33 × 10–281) and 2.69 (p-value = 1.10 × 10–158), respectively (Mann–Whitney U rank-sum test). This is consistent with the moderately skewed IgG2c>IgG1 response observed by IgG isotyping after IVAX-1 administration (Fig. 5a). We also compared the expression of the *Ifng* gene in naïve and immunized mice (Fig. 8D), which showed IFNγ was expressed by both CD4 and CD8 T cells, with log2-fold increases of 0.7 (p-value = 0.28) and 0.9 (p-value = 0.69), respectively (Mann–Whitney U rank-sum test), consistent with a robust IFNγ response in T cell recall assays (Fig. 5B). Interestingly, intracellular cytokine staining suggested IVAX-1 was not a strong inducer of antigen-specific CD8 cells (Fig. 7B), although as mentioned above, this may reflect suboptimal restimulation conditions for CD8 cells in the recall assay. Overall, these data show that the transcriptomic Th1/Th2 balance matched that defined by IgG subtyping and cytokine expression in T cell recall assays.

We also inferred cell–cell signaling interactions between different cell types using CellChat, a recently developed tool that defines intercellular signaling strengths between specified cell types in single-cell transcriptomic data. This tool has previously been used to analyze differential wound healing signaling in adult mice^38^. As shown in Fig. 8E, lymphocytes (i.e., NK/T cells, T cells, Tregs and B cells) communicate predominantly with macrophages before immunization, whereas this shifts to dendritic cells and neutrophils after immunization. The γδ T cells, B/T cell doublets and resident monocytes were excluded from the analysis because they did not meet the cutoff of 100 cells. Intra-cell type communication (e.g., T cells to T cells) is higher for lymphocytes in unimmunized mice and higher for neutrophiles and dendritic cells in immunized mice. Overall, these data provide a useful first look at the transcriptomic and cellular interactome induced by IVAX-1 at a single time point. Similar studies at different time points after vaccination, and with different adjuvants for comparison are in progress.

## Discussion

In this study, we administered mice recombinant influenza virus hemagglutinin (HA) formulated with a panel of eight different TLR agonist adjuvants administered without or with the squalene-in-water emulsion, AddaVax. A major aim was to identify adjuvants able to induce the broadest Ab response across different HA subtypes and drift variants using the microarray approach. We are cognizant that not all conformation-dependent epitopes will be available for recognition on the HA monomer microarray and may underrepresent the full polyclonal response. Nevertheless the data do reveal a performance hierarchy of the different adjuvants.. In the absence of AddaVax, most TLR agonists tested induced excellent breadth for H5 variants, while cross-reactivity for the more distantly-related H1 was confined to five of the eight adjuvants with a range of breadths induced (Fig. 1 and Table 2). AddaVax induced excellent breadth for H5, both alone and when combined with TLR agonists. Cross-reactivity for H1 and other subtypes by TLR agonists was also improved by combining with AddaVax, both in terms the number of formulations able to induce heterosubtypic cross-reactivity, and breadth induced. The response to IVAX-1 (CpG/MPLA/AddaVax) was chosen for further study as the response was the most rapid and reached the greatest magnitude (Figs. 1 and 4). IVAX-1 also elicited the strongest homosubtypic responses to the HA1 portion of H5 that contains the variable head domain (Fig. 2). IVAX-1 induced both IgG1 and IgG2c secreting B cells, with a polarization to IgG2c and a dominant Th1 cytokine profile in T cell recall assays (Fig. 5).

A key observation was IVAX-1 induced higher numbers of antigen-specific plasmablasts than the sum of the numbers when CpG/AddaVax or MPLA/AddaVax were used separately (Fig. 6), suggesting a synergistic rather than additive effect. Unlike other adjuvants such as complete Freund’s adjuvant or Titermax, IVAX-1 can be used for boosting without inducing lesions or other adverse events (data not shown). IVAX-1 can also be administered intranasally, and elicits IgA in lung and nasal washes (Fig. 7); the IgA profile displayed a cross-reactivity pattern that was identical to the IgG profile induced by the same route or by s.c. administration. Despite observing heterosubtypic cross-reactivity by antibodies, T cell responses in B6 mice administered H5 were H5-specific and did not show heterosubtypic cross-reactivity for H1 or H7. We speculate this is most likely related to a paucity of shared sequences in these HA subtypes capable of being presented by I-A^b^, the only class II MHC allele available in C57Bl/6 mice for presentation, although this has to be tested experimentally. Finally, we performed a preliminary sc mRNA seq study. In an unbiased analysis of the data, of note was elevated expression of *Ighg2c*, *Ighg2b* and *Ighg1* genes in immunized mice relative to naïve mice, consistent with the IgG subtyping by serology and flow cytometry.

The technology for producing influenza vaccines had changed little since the 1930’s. Virus is propagated in fertilized chicken eggs and inactivated using propriolactone, followed by detergent extraction to enrich the lipid membrane fraction containing the integral HA and NA antigens (so-called ‘split’ vaccines). Although alternative vaccines made from tissue culture-derived virus (Flucelvax) or recombinant HA (Flublok), are widely available, > 80% of seasonal influenza vaccines used in the U.S. are still made in eggs. Most seasonal influenza vaccines are also administered without adjuvant, in part because inactivated virus vaccines retains some inherent immunogenicity^57,58^. Effectiveness is likely related to pre-existing immunity which is easier to boost than a primary response. Protection is short-lived, in part because bone marrow plasma cells quickly wane^59^. FluAd (Seqirus), currently the only adjuvanted seasonal vaccine, is a tetravalent inactivated split virus vaccine administered in MF59^60^, a squalene-in-water emulsion that is very similar to AddaVax^61^. The Ab response to FluAd is broader than non-adjuvanted equivalents^60,62,63^. Yet, this vaccine is administered only to the elderly (> 65 years), where its improved immunogenicity is used to overcome immune senescence rather than for increased breadth or other benefits. Adjuvants play an important role in pandemic preparedness by accelerating the response, enhancing immunogenicity and breadth, and dose-sparing of vaccine^64,65^. Early efforts at adjuvanting a pre-pandemic H5N1 vaccine using alum (aluminum salts such as aluminum hydroxide, which until 2017 was the *only* adjuvant available for human use) failed to elicit sufficient neutralization^66^. However, the use of modern emulsions has led to the licensure of several pre-pandemic vaccines, including Q-Pan (ID Biomedical Corporation of Quebec), a monovalent split H5N1 vaccine, and Pandemrix (GlaxoSmithKline) monovalent split H1N1 vaccine, both adjuvanted with AS03 (GSK), a squalene-in-water emulsion with α-tocopherol^67^.

A small but growing number of pattern recognition receptor (PRR) agonists are being used in licensed vaccines. These agonists represent a new generation of vaccine adjuvants, and can be classified according to the particular PRR signaling pathway they act through, i.e., (a) toll-like receptor (TLRs), (b) nucleotide-binding oligomerization domain-like (NOD) receptors, and c) stimulators of the cGMP-AMP Synthase STimulator of INterferon Genes (cGAS-STING) pathway. Adjuvant system 4 (AS04) from GSK, which comprises traditional alum combined with monophosphoryl lipid A (MPLA), a synthetic form of the TLR4-binding domain of lipopolysaccharide (LPS)^68^, is currently used with the approved vaccines against HPV and HBV, Cervarix^69^ and Fendrix^70^, respectively. Shingrix, an approved vaccine against shingles (herpes zoster virus) is adjuvanted with AS01_B_, which consists of MPLA and the natural saponin surfactant, Quil-A (QS-21)^71^. Synthetic oligodeoxynucleotides containing unmethylated cytosine-guanine dinucleotide motifs (ODN CpG) exerts adjuvant effect through TLR9^72,73^ and has recently been approved as adjuvant for the recombinant HBV vaccine, Heplisav-B^74,75^. PRR agonists are yet to be approved for split inactivated seasonal influenza virus vaccines, despite studies showing there may be several benefits, including increasing magnitude, breadth and duration of the response. These benefits have been reviewed extensively in recent years^64,76–78^.

There is also increasing awareness that combining adjuvant components also increases the efficacy of vaccines though enhanced stimulation of APCs and lymphocytes^79–85^. In this study, we saw a synergistic effect on Ag-specific plasmablast numbers when combining CpG and MPLA. Other studies have shown CpG + MPLA stimulate the induction of IL12 from dendritic cells, while neither agonist was effective when used alone^82^. The same group also showed single-dose administration of an inactivated split influenza vaccine with CpG and MPLA led to improved homo- and hetero-subtypic cross-protection compared to vaccine administered with CpG or MPLA alone^83^. Consistent with the findings reported here, CpG + MPLA induced IgG2c (Th1) polarization. The effects of CpG and MPLA alone were more discrepant; in our hands, CpG failed to induce a significant Ab response after a single dose, and MPLA induced both isotypes (IgG2c>IgG1), while Ko et al.^83^ reported CpG induced IgG2c and MPLA induced IgG1 (Th2). These discrepancies may have been caused by the different antigen preparations used (recombinant protein used here, vs. split inactivated virus) or the doses of antigen or TLR a administered. A general consensus is both CpG and MPLA engender a Th1-biased response in B6 mice and humans^86,87^. The molecular basis of CpG + MPLA synergy has been hypothesized to arise from activation of both MyD88 and TRIF signaling pathways^88,89^.

In this study we used recombinant HA proteins, rather than split inactivated virus, to evaluate different adjuvants. Recombinant proteins may offer several advantages over current egg-based methods, including more rapid manufacture, flexibility in design, and avoidance of adaptation to growth in eggs. HA stalk-focused immunization strategies to overcome antigenic drift are also readily amenable to recombinant protein approaches^90^. Currently, Flublok is the only approved recombinant HA-based seasonal vaccine, comprised of HA proteins from four influenza viruses expressed in insect cells. Flublok is administered without adjuvant, although immunogenicity is achieved by using a threefold higher dose of each HA protein, i.e., 45 μg compared to ~ 15 μg for a typical split vaccine^64^. In our hands, doses of 2.5 μg H5 protein was unable to elicit an antibody response in B6 mice without adjuvant although high doses may overcome this. We also used a single dose regimen. Although MN and HI assays were performed on the plasma samples collected in the adjuvant screen herein, we saw no virus neutralization (data not shown), consistent with our previous studies^27^ and other data (Fig. 7A) that show boosting is required to achieve virus neutralization, at least with the antigen doses used here.

Overall, the studies described here are consistent with the notion that adjuvanted recombinant HA proteins are a possible path toward broader vaccine coverage than is currently achieved with non-adjuvanted inactivated vaccines, particularly if used in conjunction with multivalent antigens^27^. Achieving a true ‘universal’ vaccine will be challenging using adjuvants alone, without additional stalk directed immunization strategies to target conserved epitopes. Intuitively, cross-reactivity between variable epitopes is likely to be less effective than between conserved regions, as substitutions of just a few key amino acids in an epitope can abrogate neutralization. We speculate this may be offset by the use of adjuvants, which, by driving affinity maturation, may lead to higher affinity nAbs better able to tolerate substitutions than those generated by non-adjuvanted vaccines. This has the added benefit of exploiting the natural immunodominance of the head domain during vaccination and natural infection.

## Supplementary Information

Supplementary Information.
