# Supplementary material for: Magnitude and breadth of antibody cross-reactivity induced by recombinant influenza hemagglutinin trimer vaccine is enhanced by combination adjuvants
Source: Sci Rep. 2022 Jun 2;12:9198. doi: 10.1038/s41598-022-12727-y (PMC9163070; doi:10.1038/s41598-022-12727-y)
Supplement: Supplementary file 1 — Supplementary Information. [file 41598_2022_12727_MOESM1_ESM.docx]

**Supplementary Figures**

**Supplementary Figure S1. Correlation of protein microarray IgG profiles in mice administered monomeric or trimeric H5 in IVAX-1.** C57Bl/6 mice received a single dose of 2.5μg H5 protein (A/Vietnam/1203/04) as monomers from Sinobiological (N=3 mice) or trimers from Krammer lab (N=5 mice) in IVAX-1 adjuvant via s.c. route. Shown is a scatter plot of mean array signal intensities on d28 post immunization, with each spot representing a different full-length HA monomer on the array spanning H1 through H18 (N=117 different proteins). Orange, H5 drift variants (homosubtypic cross-reactivity); grey, H1 variants (an example of heterosubtypic cross-reactivity); blue, all other HA subtypes.

**
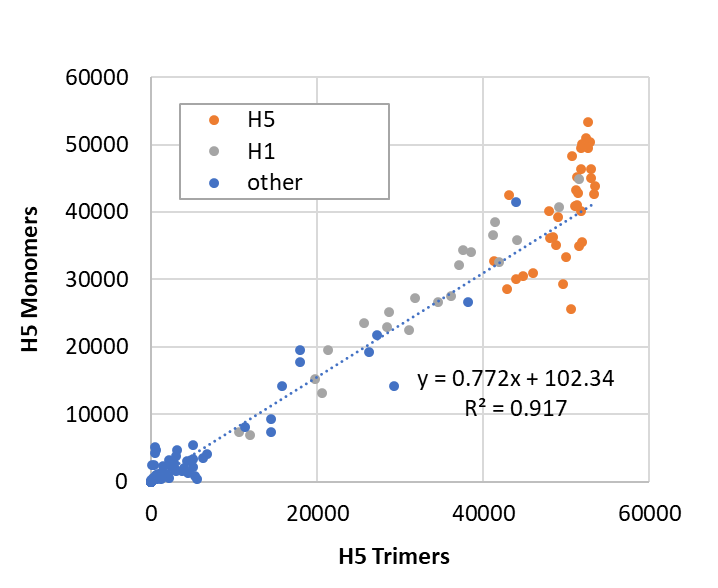
**

**Supplementary Figure S2. A)** Immunization and bleed schedule for mice in groups 1-18 (Table 1); **B)** Mouse body weights (mean +/- SD; N=4 per group) expressed as % of original body weight.

**
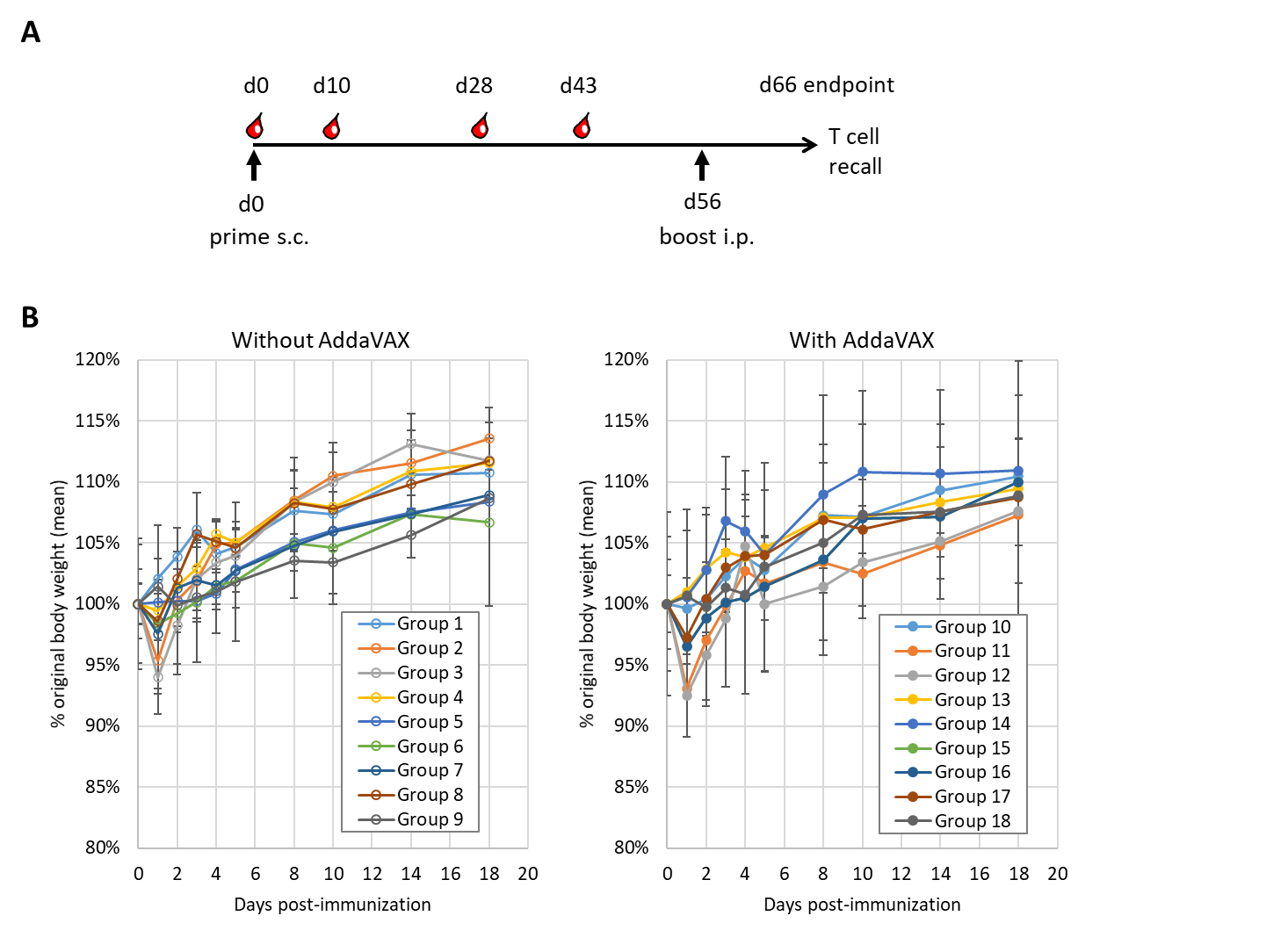
**

**Supplementary Figure S3. Celltype model training and doublet detection.** **A)** Confusion matrix of support vector machine training. The numbers in each element of the matrix indicates the fraction of cells with the correct label that were called in each predicted label category; **B)** Doublet detection scores generated by scrublet; **C)** and **D)** Expression of Cd3e and Cd79a in each cell type, respectively. The B/T cell doublet cluster is expressing both markers.

**Supplementary Figure S4.** Enhanced volcano plot comparison between all immunized and non-immunized cells after downsampling. IgG transcripts show large Log2 fold changes (FC) that are also statistically significant.

**
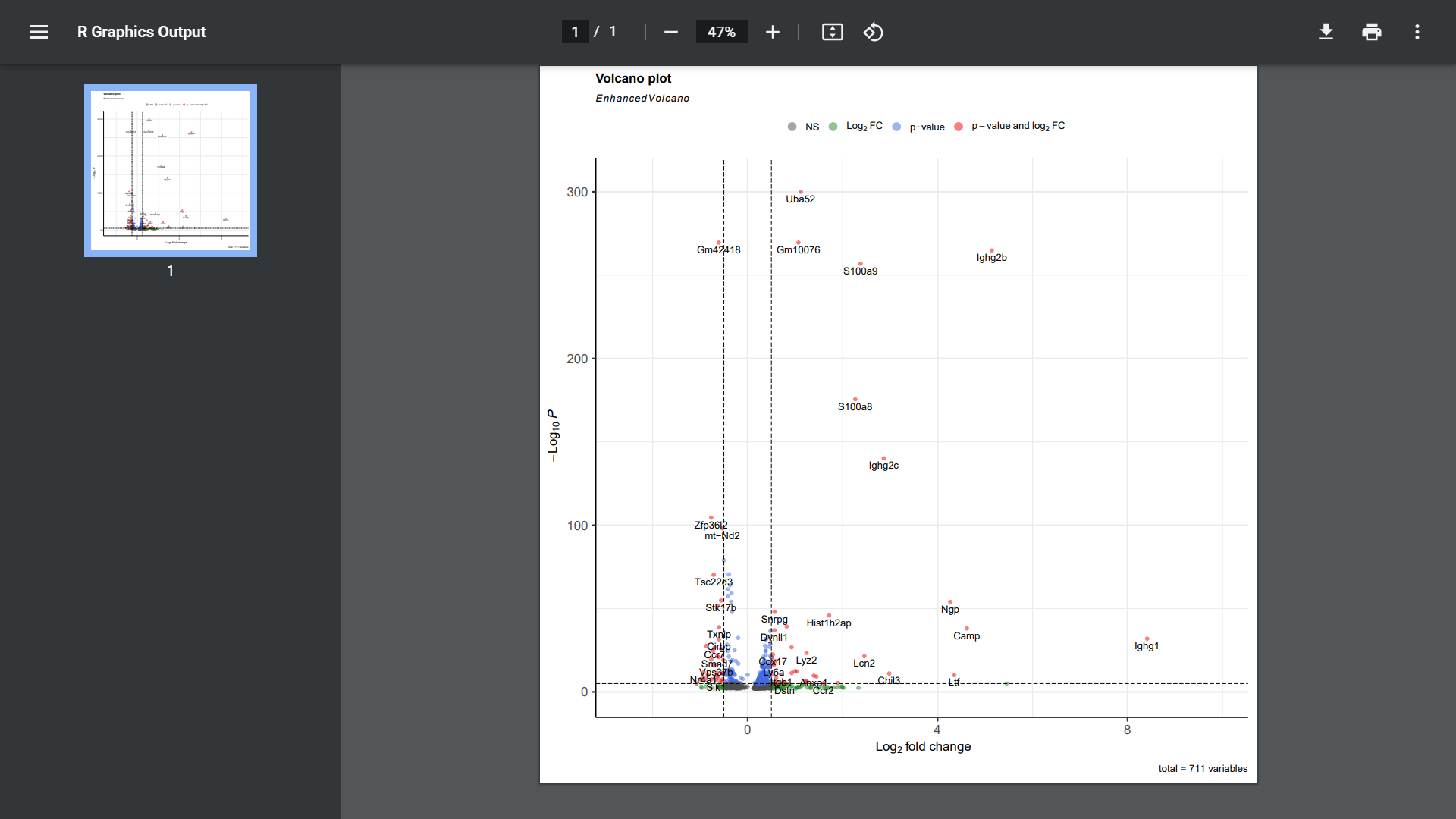
**

**Supplementary Table 1**

Significance of antibody responses to H5 administered in different adjuvants. Array profiles against HA0 (full length) and HA1 fragments of H5 variants (Figs. 1 and 2, respectively) were made using Kruskal Wallis Dunn's multiple comparison test, ****P < 0.0001; ***P ≤ 0.001; **P ≤ 0.01; *P < 0.05. Blank cells, not significant.
